# Palovarotene Action Against Heterotopic Ossification Includes a Reduction of Local Participating Activin A‐Expressing Cell Populations

**DOI:** 10.1002/jbm4.10821

**Published:** 2023-10-19

**Authors:** Christina Mundy, Lutian Yao, Kelly A. Shaughnessy, Cheri Saunders, Eileen M. Shore, Eiki Koyama, Maurizio Pacifici

**Affiliations:** ^1^ Translational Research Program in Pediatric Orthopaedics, Division of Orthopaedic Surgery The Children's Hospital of Philadelphia Philadelphia PA USA; ^2^ Department of Orthopaedics The First Hospital of China Medical University Shenyang China; ^3^ Departments of Orthopaedic Surgery and Genetics, Perelman School of Medicine University of Pennsylvania Philadelphia PA USA

**Keywords:** ACTIVIN A, ENDOCHONDRAL OSSIFICATION, FIBRODYSPLASIA OSSIFICANS PROGRESSIVA, HETEROTOPIC OSSIFICATION, PALOVAROTENE, PROGENITOR CELLS, RETINOID AGONISTS

## Abstract

Heterotopic ossification (HO) consists of extraskeletal bone formation. One form of HO is acquired and instigated by traumas or surgery, and another form is genetic and characterizes fibrodysplasia ossificans progressiva (FOP). Recently, we and others showed that activin A promotes both acquired and genetic HO, and in previous studies we found that the retinoid agonist palovarotene inhibits both HO forms in mice. Here, we asked whether palovarotene's action against HO may include an interference with endogenous activin A expression and/or function. Using a standard mouse model of acquired HO, we found that activin A and its encoding RNA (*Inhba*) were prominent in chondrogenic cells within developing HO masses in untreated mice. Single‐cell RNAseq (scRNAseq) assays verified that *Inhba* expression characterized chondroprogenitors and chondrocytes in untreated HO, in addition to its expected expression in inflammatory cells and macrophages. Palovarotene administration (4 mg/kg/d/gavage) caused a sharp inhibition of both HO and amounts of activin A and *Inhba* transcripts. Bioinformatic analyses of scRNAseq data sets indicated that the drug had reduced interactions and cross‐talk among local cell populations. To determine if palovarotene inhibited *Inhba* expression directly, we assayed primary chondrocyte cultures. Drug treatment inhibited their cartilaginous phenotype but not *Inhba* expression. Our data reveal that palovarotene markedly reduces the number of local *Inhba*‐expressing HO‐forming cell populations. The data broaden the spectrum of HO culprits against which palovarotene acts, accounting for its therapeutic effectiveness. © 2023 The Authors. *JBMR Plus* published by Wiley Periodicals LLC on behalf of American Society for Bone and Mineral Research.

## Introduction

Heterotopic ossification (HO) consists of de novo formation and local accumulation of extraskeletal bone—usually endochondral in origin—in affected anatomical sites at the expense and damage of resident structures, including muscles and connective tissues.^(^ ^1^, ^2^ ^)^ One common form of HO is acquired, often provoked by invasive surgery, deep burns, or spinal cord injury, and can limit physical activity and mobility over time and can be painful.^(^ ^2^, ^3^, ^4^ ^)^ A rare and very severe form of HO is the genetic condition fibrodysplasia ossificans progressiva (FOP), which is caused by a gain‐of‐function mutation in the type 1 bone morphogenetic protein (BMP) receptor ALK2 encoded by *ACVR1*, hampering health and body function markedly and often leading to premature death.^(^ ^5^, ^6^, ^7^ ^)^ The exact sequences of cellular and molecular steps and mechanisms leading to onset and progression of acquired and genetic HO remain to be fully grasped and understood, but general principles have emerged and are widely acknowledged.^(^ ^2^ ^)^ Thus, a physical insult in acquired HO or a spontaneous trigger (or injury) in genetic HO would result in local inflammation, followed by mobilization of local progenitor stem cells or recruitment from surrounding sources, intervention of skeletogenic cues and factors, commitment of progenitors, and finally chondrogenesis and osteogenesis.^(^ ^2^, ^8^, ^9^, ^10^ ^)^ Despite their pathological origin, the resulting HO masses are composed of seemingly normal endochondral bone, are not tumorigenic, and expand until they come to a rest, but as indicated above, can inflict a great deal of damage, pain, and physical limitations. At present, acquired HO is clinically treated by radiotherapy and/or surgical resection, but surgery cannot be used if the HO mass is difficult to reach.^(^ ^11^, ^12^ ^)^ In addition, surgery to remove acquired HO can at times trigger another round of HO, and some patients are prone to recurrent HO episodes after surgery, rendering the clinical management of acquired HO difficult.^(^ ^2^ ^)^ HO in FOP patients is wholly inoperable because the disease is reactive and surgery leads to rapid and more severe HO.^(^ ^13^ ^)^ In sum, both acquired and genetic HO persist as challenges for both patients and clinicians.

Palovarotene is a synthetic industry‐made retinoic acid receptor γ (RARγ) agonist drug that has recently been approved for treatment of genetic HO in FOP patients by HealthCanada.^(^ ^14^ ^)^ We originally conceived the idea to use palovarotene against genetic as well as acquired HO^(^ ^15^ ^)^ based on previous findings from laboratories including ours that retinoid agonists are powerful inhibitors of chondrogenesis,^(^ ^16^, ^17^ ^)^ the first overt cell differentiation phase in HO development as indicated above. The three nuclear RAR family members—RARα, RARβ, and RARγ—are expressed in specific spatiotemporal patterns, regulate multiple developmental and physiological processes, and can cause disorders when misexpressed or mutated.^(^ ^18^, ^19^ ^)^ The RARs act as transcriptional activators when bound to retinoic acid response elements (RARE) on target genes together with endogenous physiologic ligands such as all‐*trans* retinoic acid (RA), 9‐*cis*‐RA or 13‐*cis*‐RA,^(^ ^20^, ^21^ ^)^ or to synthetic retinoid agonists such as palovarotene.^(^ ^22^ ^)^ In cells lacking endogenous natural retinoids or that are rich in cytoplasmic retinoid‐catabolizing enzymes such as CYP26 members,^(^ ^23^ ^)^ the unliganded RARs also interact with RAREs on target genes but exert transcriptional repressor function.^(^ ^24^ ^)^ Transcriptional activation and repression by RARs are fundamentally important for, and relevant to, many and diverse processes.^(^ ^21^ ^)^ Indeed, Underhill and colleagues first reported that chondrogenesis normally requires unliganded RAR repressor function and that retinoid agonists undermine this requirement and inhibit cartilage differentiation by blocking key pro‐chondrogenic pathways such as canonical pSMAD1/5/8‐mediated BMP signaling.^(^ ^25^, ^26^ ^)^ Because BMP signaling and chondrogenesis are required for HO development, the inhibition of both processes by retinoid agonists such as palovarotene accounts for the drug's anti‐HO therapeutic effectiveness.^(^ ^15^ ^)^

An important and unanticipated step that advanced understanding of HO pathogenesis was provided by recent studies revealing that the transforming growth factor β (TGF β) superfamily member activin A also has an important role in genetic HO formation.^(^ ^27^, ^28^ ^)^ Activin A is encoded by *Inhba* and mainly signals via pSMAD2/3 through ALK4 and ALK7.^(^ ^29^ ^)^ In FOP, however, mutant ALK2^R206H^ was found to respond to activin A and signal through the canonical BMP pSMAD1/5/8 pathway, eliciting a chondrogenic stimulus.^(^ ^27^, ^28^ ^)^ These important findings have led to an ongoing phase 2 clinical trial with FOP patients treated with systemic administration of an activin A neutralizing monoclonal antibody (ClinicalTrials.gov, NCT0594116). In a recent study, we discovered in mouse models that activin A is needed for acquired HO formation as well,^(^ ^30^ ^)^ exerting a chondrogenic signal through its normal pSMAD2/3 pathway shared by TGFβ members that are also notorious for their chondrogenic action.^(^ ^31^ ^)^ The data suggested that activin A is a potential therapeutic target for acquired HO as well as in FOP. Despite these felicitous and promising therapeutic advances, much remains unclear about how the anti‐activin A immunotherapy and the palovarotene pharmacologic therapy dampen HO formation. In principle, these and other HO treatments under study^(^ ^32^ ^)^ could act in entirely distinct manners against HO pathogenesis and specifically by the means they were conceived for. Alternatively, they could intersect and converge on shared culprits at some point during their anti‐HO action. To begin to tackle this important and therapeutically relevant question, we specifically investigated whether palovarotene intersected with activin A to inhibit its expression and/or action. Our data reveal that palovarotene administration markedly reduced the number of activin A‐producing/*Inhba*‐expressing cell populations present at sites of acquired HO development, expanding the drug's spectrum of action against HO and accounting for its therapeutic potency.

## Materials and Methods

### Ethics statement regarding mouse studies

All mouse studies were conducted after review and approval by the Institutional Animal Care and Use Committee (IACUC) at the Children's Hospital of Philadelphia. All animals were handled, treated, and cared for according to the approved protocol, and all procedures fully complied with the ARRIVE guidelines. CD‐1 mice were obtained from Charles River Laboratories (Wilmington, MA, USA).

### HO mouse model

Standard subcutaneous model of HO was used that we and others have shown to be effective, mild, and noninvasive previously. Briefly, growth factor‐reduced, phenol red‐free Matrigel (Corning, Corning, NY, USA) was mixed with rhBMP‐2 (0.25 μg) (GenScript Biotech Corp., Piscataway, NJ, USA) per 250 μL. Matrigel alone and Matrigel containing rhBMP‐2 were prepared on ice, and 250 μL was injected at two ventral subcutaneous sites in 6‐ to 8‐week‐old CD‐1 female mice. Starting on the day of Matrigel injection, the mice received daily administrations of vehicle or palovarotene (4 mg/kg) (Atomax, Shenzhen, China) via gavage. Female mice were used because they are easier to handle and elicit a consistent HO process indistinguishable from that in males. Control mice injected with Matrigel alone did not receive palovarotene. Subcutaneous implantations were carried out under anesthesia by inhalation of 1.5% isoflurane in 98.5% oxygen as prescribed by IACUC. HO samples were harvested at days 5, 9, and 12, fixed in 4% paraformaldehyde (PFA) overnight, and then processed for histological and immunohistochemical analyses.

### Histochemistry, immunohistochemistry, and RNAscope

Day 5, 9, and 12 HO samples were fixed overnight and then immediately processed without decalcification. Fixed samples were embedded in paraffin, and serial 5.0 μm cross sections were stained with hematoxylin and eosin for routine histology, Safranin O/Fast green to reveal cartilage, and Alizarin red to reveal mineralized matrix. Representative sections were subjected to heat‐antigen retrieval and processed for immunohistochemistry (IHC) using 1:200 dilution of rabbit activin A antibody (Thermo Fisher Scientific, Waltham, MA, USA) at 4°C overnight. Companion sections were incubated with a similar concentration of preimmune rabbit IgG (Cell Signaling, Danvers, MA, USA). After rinsing, sections were reacted with biotinylated secondary antibody, followed by 3,3′‐ diaminobenzidine (DAB) color development. RNAscope in situ hybridization was carried out using RNAscope2.5 HD Detection reagent‐RED (Advanced Cell Diagnostics, Newark, CA, USA) to visualize the spatiotemporal expression of *Sox9* (cat. no. 401051), *Acan* (cat. no. 439101), and *Inhba* (Activin beta‐A) (cat. no. 455871) in day 9 HO masses. Briefly, tissue sections were pretreated with a custom reagent and hybridized with each probe for 2 hours at 40°C in a custom oven. Signal was amplified with pre‐amplifier and multiple amplifier as per manufacturer's protocols, and final signal was detected and visualized by reaction with Fast Red substrate for about 10‐minute incubation at room temperature. Companion sections were hybridized with positive (cat. no. 313911) or negative control probes (cat. no. 310043) to assure signaling specificity. Sections were counterstained with hematoxylin, dried, and sealed with Permount.

Bright‐field images were taken with a Nikon (Tokyo, Japan) Eclipse Ci equipped with a Nikon camera operated with NIS‐Elements software.

### Quantification of histological images

Images of hematoxylin and eosin–stained sections of four randomly selected independent HO masses from different mice were opened in Fiji. Different areas of periphery and center of HO masses were selected for imaging. Cells were counted manually using the cell counter plugin. Positive staining for RNAscope and IHC images was determined using Fiji. Briefly, images were opened and converted to 8‐bit. Different areas within each HO mass were selected from four samples. Thresholding for each area of the mass was consistent. Total area and percent positive area were calculated for each defined area.

### Single‐cell transcriptome analyses

Ectopic tissue masses were harvested on day 5 from initial implantation, minced, and dissociated into single‐cell suspension by treatment with dispase (50 U/mL in Hanks' balanced salt solution) for 2 hours at 30°C. Cells were then suspended in 5% FBS/DMEM at a concentration of 700 to 1200 cells/μL. Cell suspensions were immediately delivered to the Center for Applied Genomics (Children's Hospital of Philadelphia) for determination of cell viability and sample processing. Viability was >95% in all samples. cDNA libraries of cells from each group were generated by Chromium Controller (10x Genomics Inc, Pleasanton, CA, USA), barcoded, and purified as described by the manufacturer and sequenced using a 2 × 150 paired‐end configuration on an Illumina (San Diego, CA, USA) HiSeq platform at a sequencing depth of ~400 million reads. Cell Ranger (version 3.0.2) was used to demultiplex reads, followed by extraction of cell barcode and unique molecular identifiers (UMIs). The cDNA insert was aligned to the reference mouse genome (mm10). Seurat package V3 was used for filtering, variable gene selection, dimensionality reduction analysis, and clustering standardly. Doublets or cells with poor quality (genes >6000, genes <200, or >5% genes mapping to mitochondrial genome) were excluded. Expression was naturally log transformed and normalized for scaling the sequencing depth to a total of 1 × 10^4^ molecules per cell. For the integrated data set, anchors from different data sets were defined using the FindIntegrationAnchors function, and these anchors were then used to integrate data sets together with IntegrateData. Data sets were scaled by regressing out the number of UMIs and percent mitochondrial genes. Cell cycle heterogeneity effect was also regressed out by using the Seurat Cell‐Cycle Scoring function. Statistically significant principal components were selected as input for uniform manifold approximation and projection (UMAP) plots. Different resolutions for clustering were used to demonstrate the robustness of clusters. In addition, differentially expressed genes within each cluster relative to the remaining clusters were identified using FindMarkers within Seurat. Subclustering was performed by isolating the mesenchymal and skeletogenic linage clusters using known marker genes, followed by reanalysis as described above. Gene ontology analysis was performed using the clusterProfiler package, and Gene Set Variation Analysis (GSVA) was performed using GSVA package.^(^ ^33^ ^)^ All sequence data and counts matrices have been deposited in Gene Expression Omnibus with the following accession number: GSE233843. The single cell transcriptome analyses presented here were performed concurrently with those reported in our previous study on activin A roles in HO.^(^ ^30^ ^)^

To computationally delineate the developmental progression of HO from mesenchymal to chondrogenic cells and order them in pseudotime trajectory, we performed trajectory analysis using Slingshot.^(^ ^34^ ^)^ To do so, Seurat objects were transformed into SingleCellExperiment objects. Slingshot trajectory analysis was conducted using the Seurat clustering information and with dimensionality reduction produced by UMAP.

### Cell–cell interaction analysis

Cellchat package (v0.0.2)^(^ ^35^ ^)^ was conducted to analyze the cell–cell communications in HO. A standard pipeline can be found in https://github.com/sqjin/CellChat/blob/master/vignettes. We first set ligand‐receptor interaction list in mouse and selected a customized “Secreted Signaling” as communicating ways. We then projected the gene expression data onto the protein–protein interaction (PPI) network by identifying overexpressed ligand‐receptor interactions. To obtain biologically significant cell–cell communications, probability values for each interaction were calculated by performing permutation tests. The inferred intercellular communication network of each ligand‐receptor pair and each signaling pathway was summarized and visualized by circle plots.

### Preparation, treatment, and analysis of growth plate chondrocyte cultures

Primary growth plate chondrocytes from the murine sternum and ribs were prepared from postnatal day 4 mice as described previously.^(^ ^36^, ^37^ ^)^ Briefly, sterna and ribs were initially digested in Pronase (2 mg/mL) (Roche, Basel, Switzerland) for 1 hour at 37°C to liberate soft tissue. Sterna and ribs were further digested in Collagenase D (3 mg/mL) (Roche, Basel, Switzerland) for 1.5 hours at 37°C, then Collagenase D (5 mg/mL) for 3 hours at 37°C. The cell suspension was strained through a 70‐μm strainer and diluted to a concentration of 8 × 10^5^ cells/mL in Dulbecco's modified Eagle's medium (DMEM) containing 10% fetal bovine serum (FBS) and antibiotics. Cells were plated on 12‐ and 24‐well tissue culture plates. Once confluent, beta‐glycerophosphate and ascorbic acid were added to the medium to induce chondrocyte maturation. Cultures were stained with Alcian blue (pH 1.0) after 4 and 7 days to monitor chondrogenic cell differentiation. Images were taken with a Nikon SMZ‐U microscope equipped with a SPOT insight camera and acquired with SPOT 4.0 software. For quantification, Alcian blue dye was extracted with 6 M guanidine hydrochloride overnight at room temperature. Absorbance values were measured at 595 nm.

### Gene expression analysis

Total RNA was isolated from control and treated chondrocyte cultures on days 4 and 7 using TRIzol reagent (Life Technologies, Carlsbad, CA, USA) according to the manufacturer's protocol. RNA was quantified by NanoDrop. One microgram of total RNA was reverse‐transcribed using iScript cDNA Synthesis Kit (Bio‐Rad, Hercules, CA, USA). Quantitative real‐time polymerase chain reaction (PCR) was carried out using the SYBR Green PCR Master Mix or TaqMan Gene Expression Master Mix (Life Technologies) in an Applied Biosystems 7500 according to the manufacturer's protocol. *Rn18s* was used as the endogenous control, and relative expression was calculated using the ΔΔC_T_ method. Primer information is in Supplemental Table S1. The following primers were purchased as TaqMan Gene Expression Assays: *Rn18s* (Mm03928990_g1) and *Inhba* (Mm00434339_m1).

### Statistical procedures

Statistical significance of data from experimental groups was evaluated by Student's *t* test and one‐way ANOVA followed by Tukey's multiple comparison test. Data were plotted using GraphPad Prism 7 (GraphPad Software, San Diego, CA, USA), and data points, averages, and 95% confidence intervals (CIs) are included in scatter plots presented below. A statistical difference of *p* < 0.05 was considered significant, and *p < 0.05, **p < 0.01, and ***p < 0.0001 denote degrees of statistical significance. Representative images were used to illustrate observations, and number of animals used in each experiment is specified in respective figure legend. Cell culture experiments were repeated independently at least three times, and data from a representative experiment are presented.

## Results

### Palovarotene inhibits the recruitment of progenitors into the developing HO mass

As an experimental model of acquired HO, we applied a popular approach in which a Matrigel scaffold containing recombinant human BMP2 or BMP6 is implanted at one or multiple subdermal locations in the abdominal area of adult mice.^(^ ^38^, ^39^ ^)^ Though experimental, this model mimics the development of HO observed in patients receiving local implantation of rhBMP2 to repair skeletal defects.^(^ ^40^ ^)^ An additional advantage of this model is that immediately after implantation, the scaffold is surrounded by inflammatory cells and other cell populations and becomes progressively invaded by these cells as they migrate deeper and deeper into it.^(^ ^41^ ^)^ This provides a unique opportunity to monitor and characterize the phenotype, architecture, and differentiation of the cells spatiotemporally as they take part in HO formation.

**Fig. 1:**
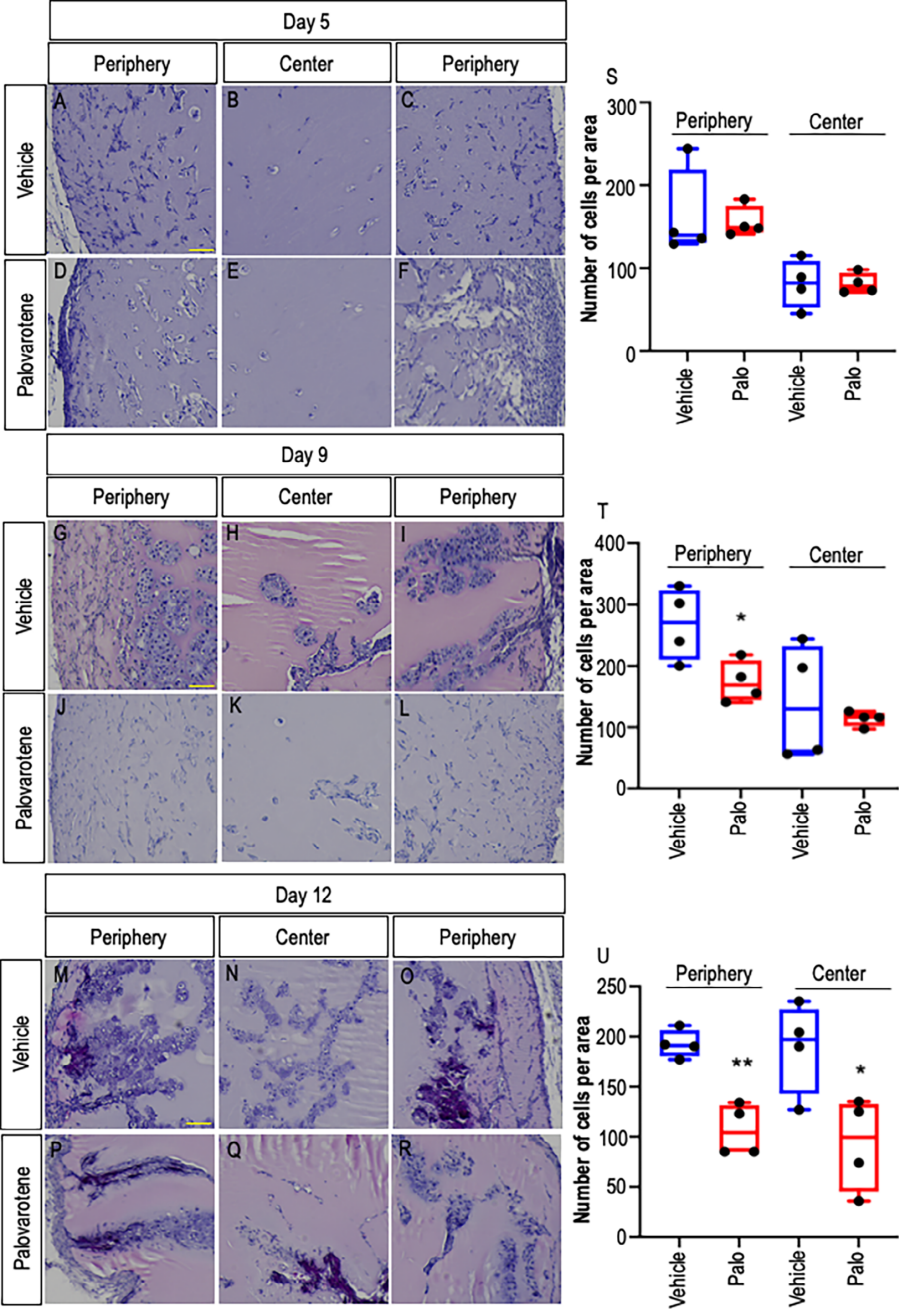
Cell accrual and infiltration are decreased by palovarotene administration. (*A–C*) Representative histological images from the periphery and center of Matrigel heterotopic ossification (HO) masses retrieved from vehicle‐receiving control mice on day 5 from implantation. (*D–F*) Histological images from the periphery and center of Matrigel HO masses retrieved from palovarotene‐treated mice on day 5 from implantation. (*G–I*) and (*J–L*) Histological images from samples retrieved from control and treated mice on day 9, respectively. (*M–O*) and (*P–R*) Histological images from samples retrieved from control and treated mice on day 12, respectively. (*S–U*) Quantification of the number of cells per area. Note the delay in cell presence and infiltration into the central portion of the HO mass after palovarotene treatment. Days 5, 9, and 12: *n* = 4 ectopic masses from control and treated mice. *p ≤ 0.05 and **p ≤ 0.01. Scale bar = 100 μm.

Accordingly, we implanted adult female mice at two abdominal subdermal sites with 250 μL Matrigel/0.25 μg rhBMP2 mixture.^(^ ^39^ ^)^ Mice were randomly divided into two groups that, starting on day 1 from implantation, received systemic administration of vehicle or palovarotene (4 mg/kg/d) by gavage as in previous studies.^(^ ^39^, ^42^ ^)^ Ectopic tissue masses were harvested on days 5, 9, and 12 from implantation and were processed for histological analysis of cell distribution, degree of scaffold immigration, and skeletogenic phenotype. In day 5 samples, many cells were present within the periphery of the Matrigel mass in control vehicle‐receiving mice, spanning a thickness of about 150 to 200 μm (Fig. 1A,C,S), whereas the center had fewer cells than the periphery (Fig. 1B,S). By days 9 and 12, the number of cells within the periphery had increased substantially (Fig. 1G,I,M,O,T,U) and the center was now rich in cells as well (Fig. 1H,N,T,U). At these stages, both periphery and center displayed cartilage tissues (see below for further characterization). In contrast, samples retrieved from palovarotene‐treated mice exhibited a slight decrease in the number of cells present at the periphery and center on day 5 (Fig. 1D,E,F,S). There was also a substantial decrease in the number of cells present at the periphery at days 9 and 12 (Fig. 1J,L,P,R,T,U) with a concurrent delay in cell invasion of the center by day 12 (Fig. 1K,Q) compared with respective controls.

**Fig. 2:**
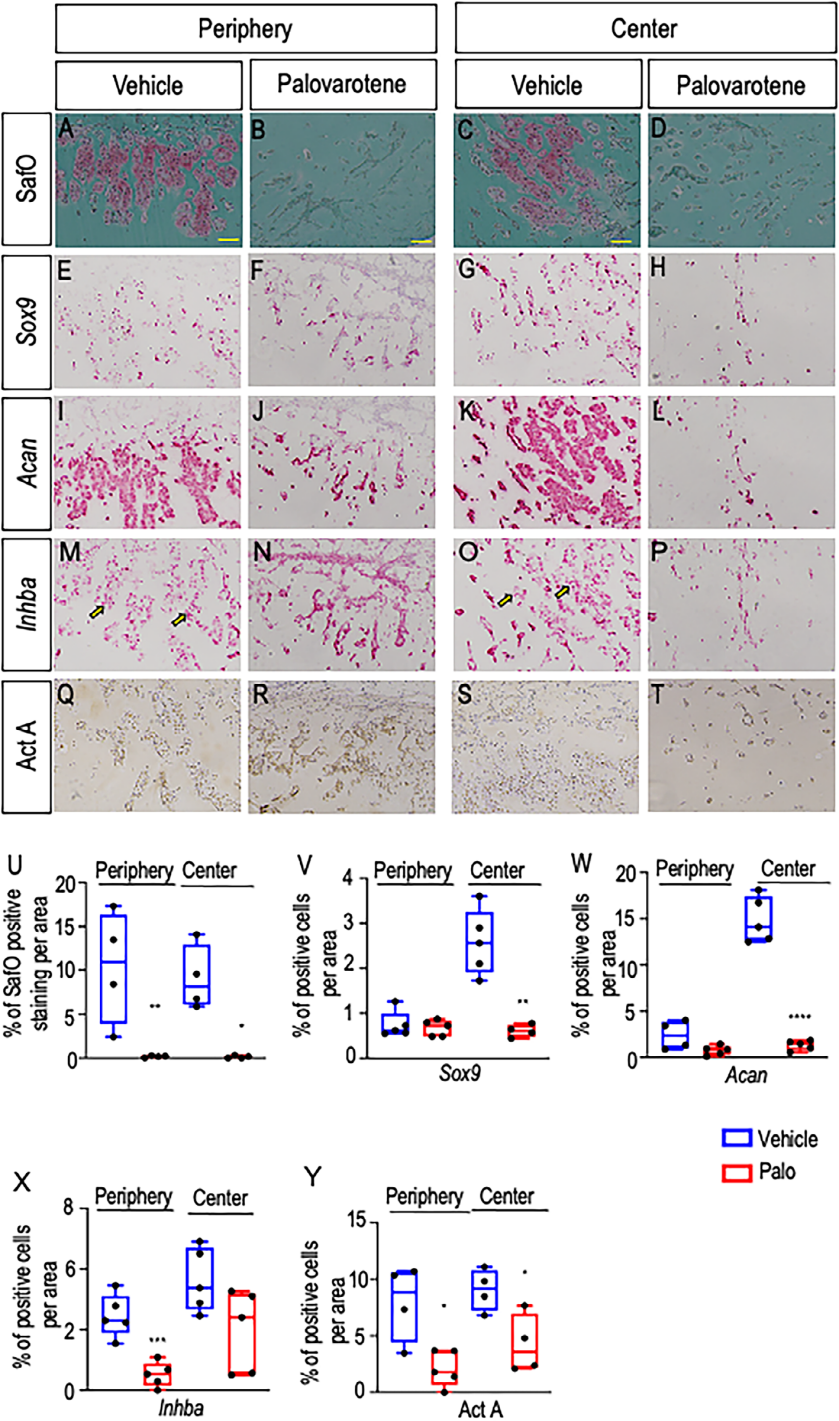
Palovarotene administration dampens cartilage formation and *Inhba*‐expressing cells. (*A–D*) Representative Safranin‐O‐stained images of peripheral and central regions of Matrigel heterotopic ossification (HO) masses retrieved from vehicle control and palovarotene‐treated mice on day 9. (*E–H*) RNAscope analysis of *Sox9* gene expression in the peripheral and central regions of companion control and treated HO masses on day 9. (*I–L*) and (*M–P*) RNAscope analysis of aggrecan (*Acan*) and *Inhba* gene expression in the peripheral and central regions of companion control and treated HO masses on day 9, respectively. (*Q–T*) Immunostaining of activin A in the peripheral and central regions of companion control and treated HO masses on day 9. (*U–W*) Quantification of *Sox9*, *Acan*, and *Inhba* gene expression. (*X*) Quantification of ActA protein expression. Note that serial sections were used for analyses of RNA and protein expression in each sample. *n* = 4–5 ectopic masses from control and treated mice, producing consistent observations. *p ≤ 0.05, **p ≤ 0.01 ***p ≤ 0.001 and ****p ≤ 0.0001. Scale bar = 100 μm.

To define the character of HO‐participating cells, we processed day 9 specimens for cartilage histochemical staining with Safranin O and for RNAscope analysis of gene expression. As expected, cartilage was evident in both periphery and center in control specimens (Fig. 2A,B) and strongly expressed typical phenotypic genes such as *Sox9* and aggrecan (*Acan*) (Fig. 2E,F,I,J,U,V). Of relevance here was the finding that the activin A‐encoding gene *Inhba* was clearly expressed by many cells in these control specimens, including cartilage cells (Fig. 2M,N,W arrows). Immunostaining with activin A antibodies elicited similar patterns (Fig. 2Q,R,X). In comparison, samples retrieved from palovarotene‐treated mice exhibited minimal Safranin O staining (Fig. 2C,D) and minimal *Sox9* and *Acan* expression, reflecting a blockage of HO (Fig. 2G,H,K,L,U,V). Interestingly, both *Inhba* expression and activin A immunostaining were markedly decreased overall but were still appreciable in the sparse cells distributed over the periphery, likely including skeletogenic progenitors (Fig. 2O,P,S,T,W,X).

### Single‐cell RNA‐seq identifies distinct cell populations during HO progression

To capture and delineate the nature of *Inhba*‐expressing cells recruited into the HO process and changes effected by palovarotene treatment, we carried out single‐cell RNA sequencing (scRNA‐seq) analysis on incipient HO masses collected on day 5. Mice implanted subcutaneously with rhBMP2/Matrigel mixture received daily doses of vehicle (termed Vehicle heretofore) or palovarotene (termed Palo heretofore) as above. As an additional control, companion mice were implanted with Matrigel without rhBMP2 (termed Matrigel‐only heretofore). On day 5, ectopic tissue masses were harvested from each mouse group, and their cells were liberated by enzymatic digestion and directly processed for scRNA‐seq (about 20,000 cells per sample).^(^ ^43^ ^)^ Cell libraries were generated by Chromium controller (10x Genomics, Inc.), barcoded, and sequenced on an Illumina HiSeq platform, and data were analyzed with Cell Ranger to obtain UMIs. We utilized the Seurat analysis package V3^(^ ^44^ ^)^ for filtering, variable gene selection and dimensionality reduction, and graph‐based clustering. Total cell numbers in the data sets obtained were: Matrigel‐only, 8728 cells; Vehicle, 5476 cells; and Palo, 7296 cells.

**Fig. 3:**
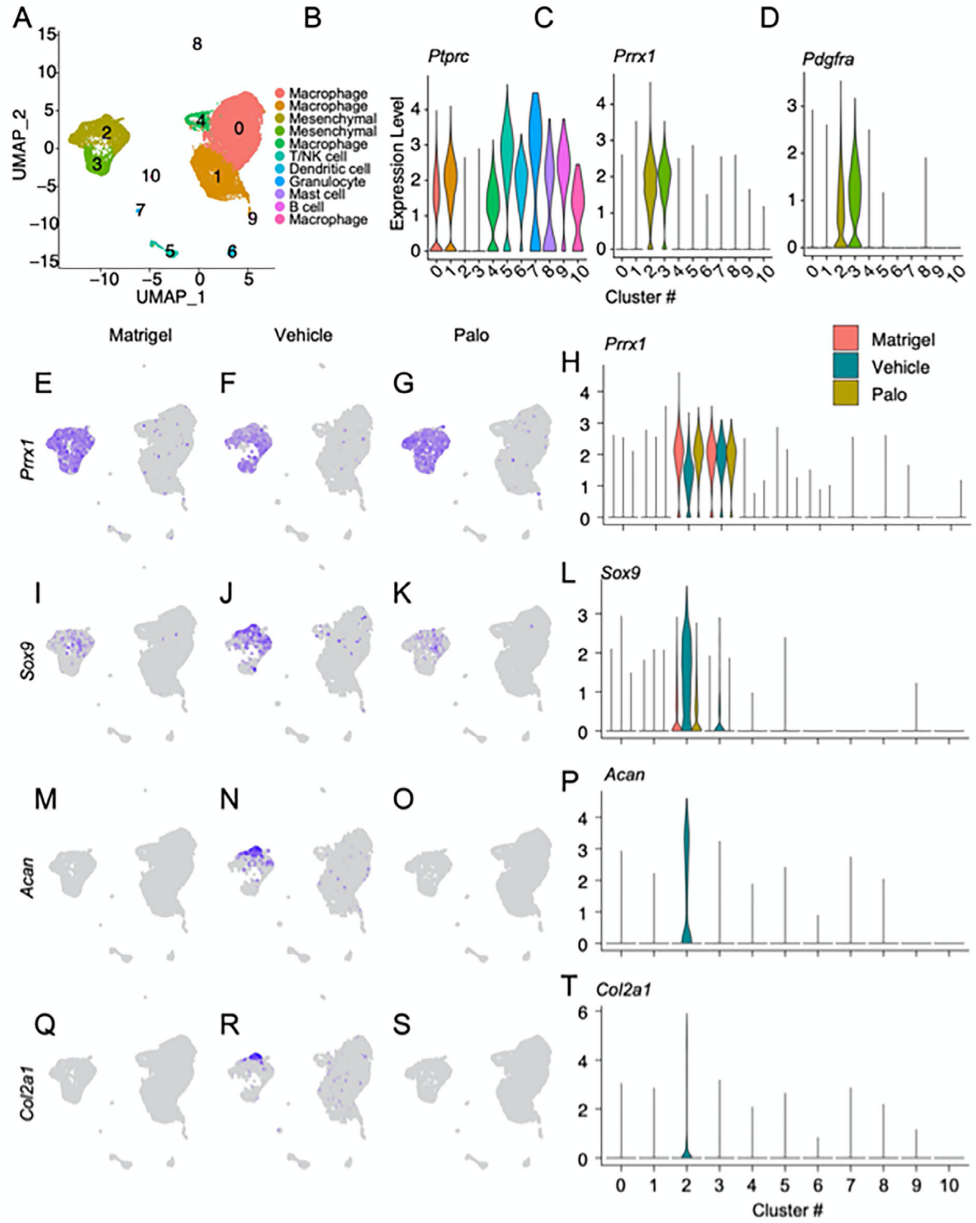
scRNA‐seq analysis of cell populations participating in heterotopic ossification (HO) development. HO tissue samples were retrieved on day 5 from mice implanted with Matrigel‐only and from companion mice implanted with Matrigel/BMP2 mixture and treated with vehicle (Vehicle) or palovarotene (Palo). (*A*) Sample contribution to cell type clusters after superimposition of UMAP data from all samples, yielding 11 major cell clusters (0–10). (*B*) Violin plots of selected genes identifying nine inflammatory and hematopoietic cell clusters sharing a typical trait, protein tyrosine phosphatase receptor type C (*Ptprc*). (*C*, *D*) Violin plots showing skeletogenic and mesenchymal cell clusters 2 and 3 that expressed characteristic traits such as platelet‐derived growth factor receptor alpha (*Pdgfra*) and/or paired‐related homeodomain transcription factor 1 (*Prrx1*). (*E–H*) UMAP plots (left) and violin plot (right) describing *Prrx1* expression that characterizes cluster 2 and 3 cells and remained largely unchanged in Matrigel‐only, Vehicle, and Palo samples. (*I–T*) UMAP plots (left) and violin plots (right) depicting expression of *Sox9* (*I–L*), *Acan* (*M–P*), and *Col2a1* (*Q–T*) among cluster 2 and 3 cells. *n* = 6 ectopic masses per each experimental group.

To perceive and define the overall composition and phenotype of the cell clusters, we first superimposed the data sets from Matrigel‐only, Vehicle, and Palo samples (Fig. 3A). Using differential expression of representative marker genes described previously,^(^ ^30^, ^45^ ^)^ we obtained and identified 11 major cell population clusters numbered 0–10 (Fig. 3A). Clusters 2 and 3 represented mesenchymal lineage cells that were characterized by expression of markers such as *Pdgfra* and *Prrx1* ^(^ ^46^, ^47^ ^)^ depicted and quantified in violin plots (Fig. 3C,D). These clusters included also some cells expressing chondrocyte and fibroblast‐like gene markers such as *Acan*, *Postn*, *Col3a1*, *Ly6a*, etc. Most of the remaining clusters contained hematopoietic and inflammatory cells identified by common expression of *Ptprc* ^(^ ^48^ ^)^ (Fig. 3B). The marker genes of these cells are shown as violin plots in Supplemental Fig. S1. Clusters 0, 1, 4, and 10 contained monocytes and macrophage lineage cells. Cluster 5 included T and natural killer cells. Cluster 6 was rich in dendritic cells. Cluster 7 included granulocytes, cluster 8 contained mast cells, and cluster 9 contained B cells (Supplemental Table S2). The presence of these various cell populations reflects the early inflammatory phase of HO development that, as pointed out above, occurs in the subcutaneous model used here as well.^(^ ^39^ ^)^

Given the main goals of the present study, we further analyzed the mesenchymal cell populations in clusters 2 and 3 (Supplemental Table S3). The Matrigel‐only samples exhibited a significant number of *Prrx1*‐expressing mesenchymal cells in both clusters (Fig. 3E,H, red color) reflecting the fact that the Matrigel scaffold itself elicits a significant host response and promotes cell recruitment and invasion. There was also an appreciable number of *Sox9*‐expressing cells within cluster 2 (Fig. 3I,L) that lacked *Acan* and collagen 2 (*Col2a1*) expression (Fig. 3M,P,Q,T), thus representing potential skeletogenic progenitors present at the very beginning of ectopic skeletal tissue formation.^(^ ^49^ ^)^ In the Vehicle samples, *Sox9*‐expressing cells as well as chondrocytes expressing *Acan* and *Col2a1* had become numerous (Fig. 3J,N,R) and after quantification by violin plots (Fig. 3L,P,T, green color). The chondrogenic cells and chondrocytes were largely present within the upper half of cluster 2 (Fig. 3N,R) as compared to the more primitive *Sox9*‐expressing cells present also in the lower half (Fig. 3J). In line with the reduction in HO initiation, the Palo samples were characterized by a sharp drop in the number of *Sox9‐, Acan‐*, and *Col2a1*‐expressing cells, visible in the UMAP plots (Fig. 3K,O,S) and violin plots (Fig. 3L,P,T, yellow color). The number of mesenchymal *Prrx1*‐expressing cells remained largely unaffected (Fig. 3G).

To gain insights into involvement and possible modulation of protein signaling and cell–cell interactions, we subjected the above data sets to gene set variation analysis (GSVA)^(^ ^50^ ^)^ of KEGG pathway database and Hallmark gene sets.^(^ ^51^ ^)^ We found that the Vehicle group displayed a significant and distinct upregulation of glycolysis and hypoxia pathways (Supplemental Fig. S2, yellow box). These are known to be linked to chondrogenic cell differentiation and chondrocyte function^(^ ^52^, ^53^ ^)^ and were both downregulated in the Palo group (Supplemental Fig. S2, yellow box). CellChat signaling analysis predicted several interactions among the various cell populations present in the HO masses (Supplemental Fig. S3). Interestingly, the palovarotene group was predicted to exhibit a significant drop in interactions among various cell populations including mesenchymal and chondrogenic cells, reflecting palovarotene's ability to dampen cartilage formation.

### *Inhba* ‐expressing cell populations are reduced by palovarotene administration

**Fig. 4:**
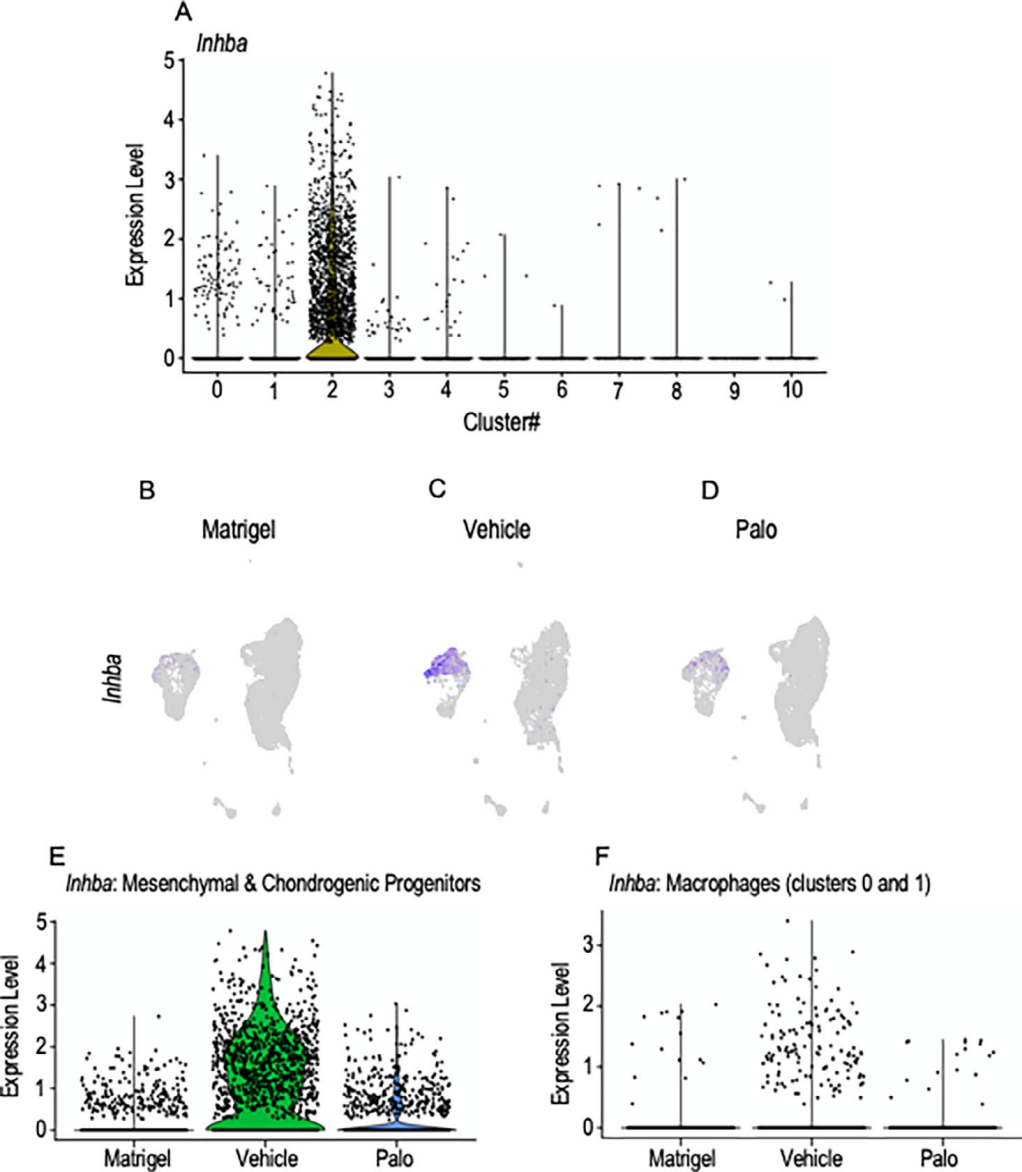
*Inhba*‐expressing chondrogenic and macrophage populations decrease after palovarotene administration. (*A*) Violin plots of superimposed data sets from Matrigel‐only, Vehicle, and Palo samples showing that *Inhba* expression prominently characterized cluster 2 cells and to some extent cluster 0 and 1 cells. (*B–D*) UMAP plots depicting *Inhba*‐expressing cells that were most prominent in cluster 2 and were increased in Vehicle samples (*C*) compared with Matrigel‐only and Palo samples (*B* and *D*, respectively). (*E*) Violin plots of *Inhba*‐expressing cells present in cluster 2 in Matrigel‐only, Vehicle, and Palo samples. (*F*) Violin plots of *Inhba*‐expressing macrophages present in clusters 0 and 1 in Matrigel‐only, Vehicle, and Palo samples. *n* = 6 ectopic masses per each experimental group.

Next, we examined cells expressing *Inhba* within clusters 0 to 10. Violin plots of superimposed data sets from Matrigel‐only, Vehicle, and Palo samples showed that the bulk of *Inhba*‐expressing cells resided in cluster 2 (Fig. 4A). UMAP plots of each data set revealed that some *Inhba*‐expressing cells were present in Matrigel samples, being more obvious in the lower half of cluster 2 (Fig. 4B), where primitive chondrogenic cells resided (Fig. 3I). Their number had much increased in the Vehicle samples and spanned both the lower and upper portions of cluster 2 (Fig. 4C), with the upper portion containing more mature chondrogenic cells as indicated above (Fig. 3K,O,S). The reduction in HO initiation and progression in Palo samples was accompanied by a sharp overall decrease in *Inhba*‐expressing cells (Fig. 4D). These significant shifts in *Inhba*‐expressing cell numbers became obvious when quantified in violin plots (Fig. 4E). In addition to cells in cluster 2, there was a clear number of *Inhba*‐expressing cells in clusters 0 and 1 representing macrophages (Fig. 4A) that were identified by expression of markers such as *Cd68*, *Adgre1*, *Csf1r*, and *C1qa* ^(^ ^30^ ^)^ and are well known for expressing *Inhba*.^(^ ^54^ ^)^ Violin plot analysis showed that the number of *Inhba*‐expressing macrophages was markedly higher in Vehicle samples compared with both Matrigel‐only and Palo samples (Fig. 4F). Their reduction after palovarotene administration likely reflects the notable anti‐inflammatory properties of retinoid agonists.^(^ ^55^, ^56^ ^)^ Because macrophages promote HO on their own,^(^ ^57^ ^)^ their reduction likely contributed to dampening HO.

**Fig. 5:**
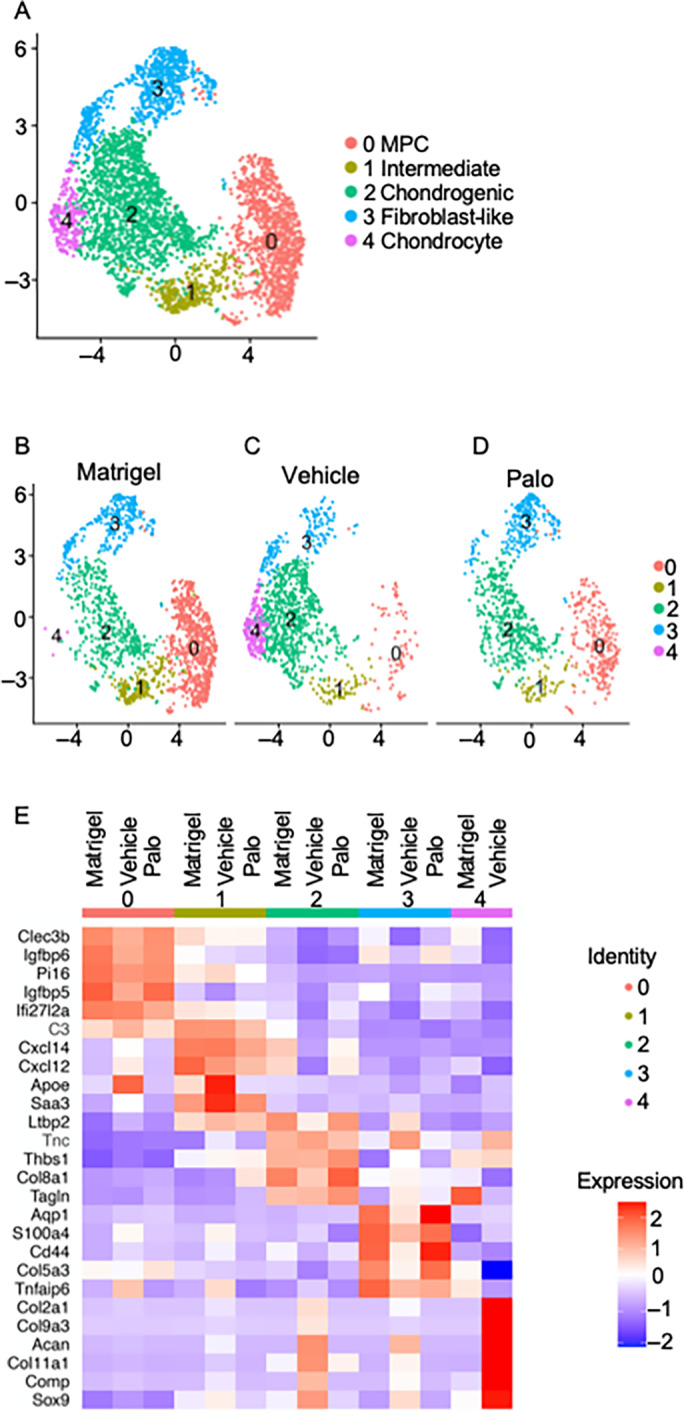
Further dissection of *Inhba*‐expressing cells yields five subclusters. Data sets of clusters 2 and 3 extracted from Matrigel‐only, Vehicle, and Palo samples shown in Figure 3 were subjected to a new round of analysis on their own. (*A*) Superimposition of UMAP data from all samples yielding five main subclusters (0–4) that based on differential gene expression were identified as mainly representing: subcluster 0, *Cd34+* mesenchymal progenitor cells (MPC); subcluster 1, progenitors with an intermediate phenotype geared to differentiation; subcluster 2, chondrogenic cells; subcluster 3, *Cd44+* fibroblast‐like cells; and subcluster 4, chondrocytes. (*B–D*) Individual UMAP data set of Matrigel‐only, Vehicle, and Palo samples. (*E*) Heat map of differentially expressed genes depicts the phenotypic diversity of cells in each of the five subclusters. Note that there were no cells to analyze in subcluster 4 Palo samples.

To discern in greater detail the nature of mesenchymal and chondrogenic populations and their modulations by drug treatment, we extracted cluster 2 and 3 data sets from Matrigel‐only, Vehicle, and Palo samples above and subjected them to a new round of Seurat analysis on their own. We obtained a more granular cell separation into five UMAP subclusters numbered 0 to 4 visible in the overlapped plot (Fig. 5A) and each separate plot (Fig. 5B–D). The subclusters were readily distinguishable based on differential gene expression (Fig. 5E) as were their dynamic behaviors under the experimental conditions used. It was already notable at this level of analysis that the relative sizes of subclusters 0 and 1 were substantial in Matrigel‐only samples, had decreased sharply in Vehicle samples but were substantial again in Palo samples (Fig. 5B–D). Also, subcluster 4 was only clear in Vehicle samples but was barely detectable in the Matrigel‐only samples and was essentially undetectable in the Palo samples (Fig. 5B–E).

**Fig. 6:**
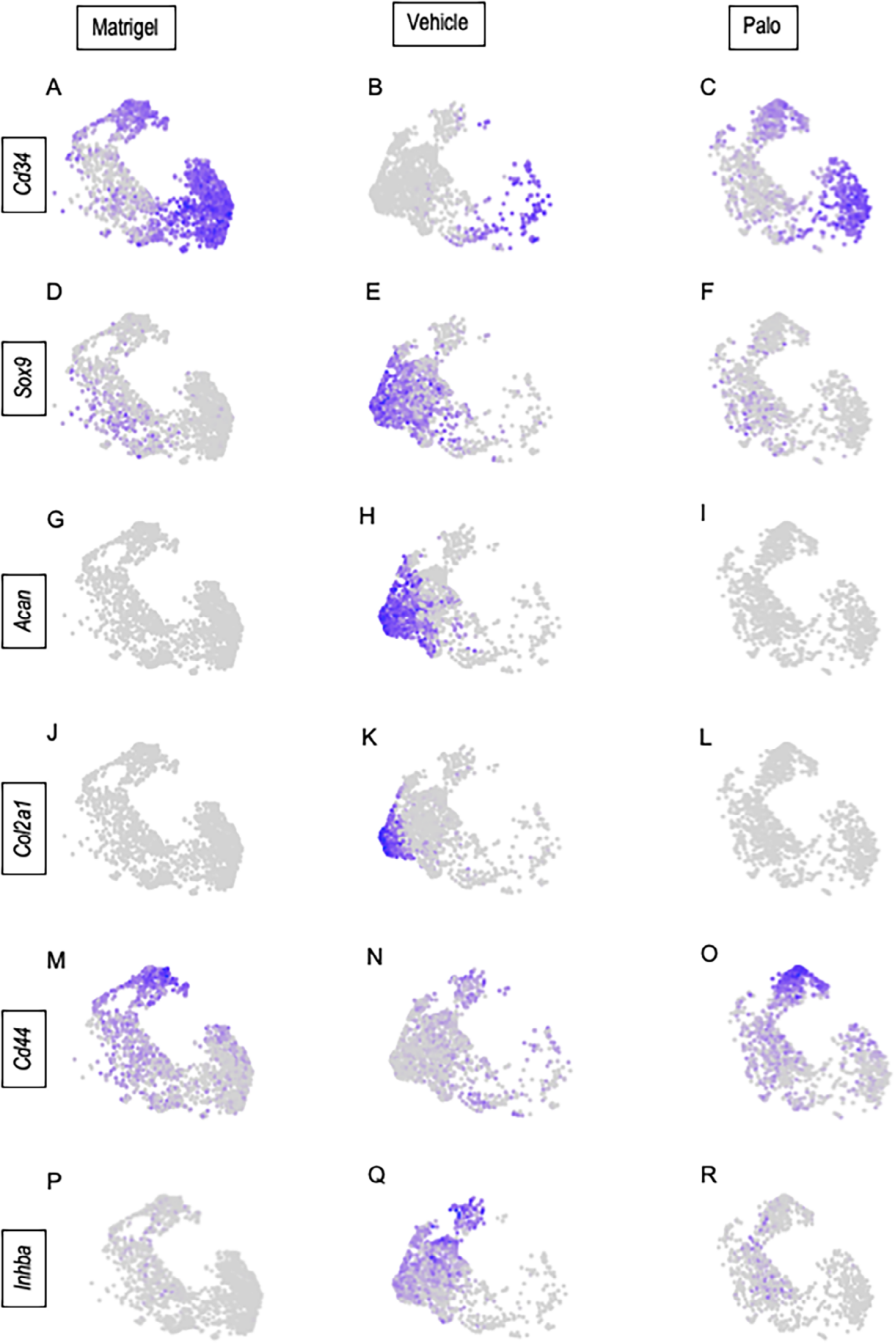
Phenotypes of cell populations present in the subclusters. UMAP plots from the individual Matrigel‐only, Vehicle, and Palo samples display the distribution of cell types in the five subclusters based on expression of typical marker genes: (*A–C*) *Cd34*; (*D–F*) *Sox9*; (*G–I*) *Acan*; (*J–L*) *Col2a1*; (*M–O*) *Cd44*; and (*P–R*) *Inhba*.

**Fig. 7:**
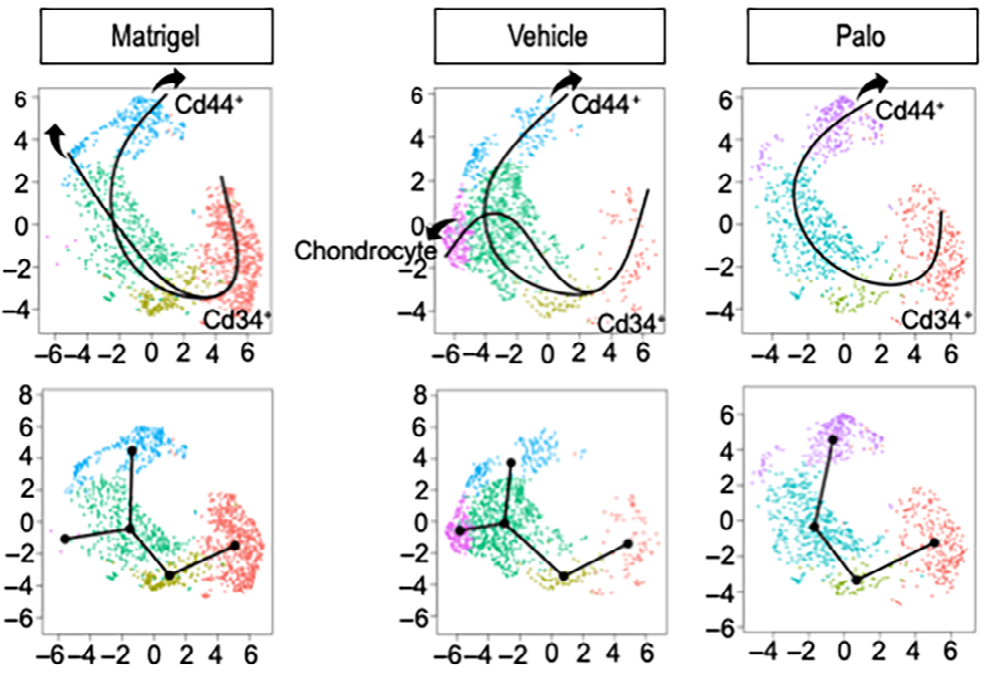
Pseudotime cell developmental trajectories based on differential gene expression patterns in the three experimental conditions. The trajectory predicts a developmental progression starting in subcluster 0 and advancing through subclusters 1 and 2 and ending in subcluster 4, with a clear bifurcation toward chondrocyte subcluster 4 appreciable in the Vehicle samples only.

Phenotypically, subcluster 0 contained cells expressing markers of mesenchymal progenitor cells (MPC) such as *Cd34* (Fig. 6A–C).^(^ ^58^ ^)^ As pointed out above, the size of this subcluster was substantial in Matrigel‐only samples and had decreased sharply in Vehicle samples but was substantial again in Palo samples (Fig. 6A–C). This trend suggested that the mesenchymal cells were committing and differentiating under BMP2 regimen, likely toward chondrogenesis, and this path was obstructed and reversed by palovarotene. Cells in subcluster 1 expressed genes that are typical of cells committed to diversification and differentiation, including *Cxcl12* and *Cxcl14* (Fig. 5E), and likely participating in chondrogenesis as well (labeled as “intermediate” in Fig. 5A). Cells in subcluster 2 had a typical *Sox9*‐expressing chondrogenic phenotype (Fig. 6D–F), were more numerous in the Vehicle samples, and were lower again in Palo samples (Fig. 6F). Subcluster 4 was composed of *Acan‐/Col2a1*‐expressing chondrocytes and was only clear in the Vehicle samples (Fig. 6G–L). Lastly, subcluster 3 was demarcated by traits such as the cell surface receptor *Cd44*, sharing this trait with progenitor and fibroblastic cells.^(^ ^59^ ^)^ The *Cd44+* cells were obvious in Matrigel‐only and Palo samples, but their number had dropped in Vehicle samples (Fig. 6M–O), a change that remains to be explained. Within the above phenotypic patterns, *Inhba*‐expressing cells constituted the bulk of subclusters 2–4 in Vehicle samples (Fig. 6Q), thus overlapping chondrogenic cells (Fig. 6E,H,K). Their number was markedly lower in both Matrigel‐only and Palo samples (Fig. 6P,R), the latter in conjunction with HO inhibition. The above trends were visualized and verified by developmental trajectory and pseudotime analyses^(^ ^60^, ^61^ ^)^ that revealed a developmental trajectory initiating in mesenchymal cell‐rich subcluster 0, transitioning through subclusters 1 and 2, progressing toward subcluster 3, and bifurcating toward the chondrocyte‐rich subcluster 4 in Vehicle as well as Matrigel‐only samples (Fig. 7). The complete absence of bifurcation in Palo samples reaffirms the drug's potency against chondrogenesis and chondrocyte phenotypic expression.

### Palovarotene does not reduce *Inhba* expression in chondrogenic cells in vitro

**Fig. 8:**
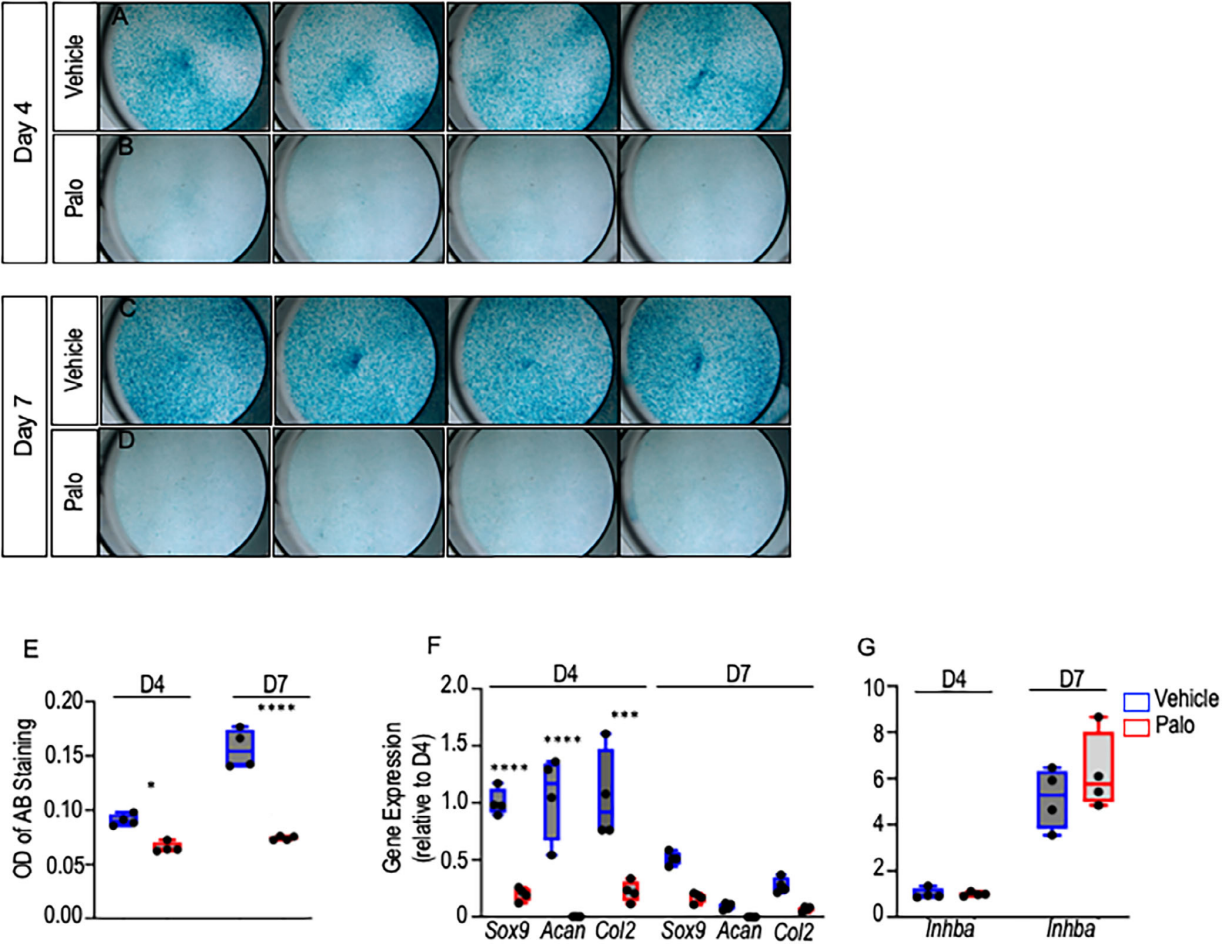
Palovarotene does not affect *Inhba* expression in cultures of chondrogenic cells. (*A–E*) Day 4 and day 7 images and quantification of Alcian blue staining of P3 chondrocytes treated with Vehicle or palovarotene (50 nM). (*F*) Expression of chondrogenic genes *Sox9*, *Acan*, and *Col2a1* and (*G*) *Inhba* in days 4 and 7 Vehicle versus palovarotene‐treated cultures. *p ≤ 0.05, ***p ≤ 0.001, and ****p ≤ 0.0001. *n* = 4 independent experiments and data are presented as means ± SD.

Data above indicate that palovarotene administration resulted in a significant decrease in chondrogenic populations expressing *Inhba*/activin A present in developing HO masses. To determine whether palovarotene exerted direct effects on *Inhba* expression itself, we tested it in cultures of chondrogenic cells. Accordingly, growth plate chondrocytes were isolated from P3 ribs and sterna and were respectively reared in monolayer culture as described.^(^ ^36^, ^37^ ^)^ Starting on day 1, cultures were given vehicle or palovarotene (50 nM) added to the culture medium, a dose that maximally decreases chondrogenesis.^(^ ^42^ ^)^ Fresh medium and drug were given every other day. Cultures were harvested on days 4 and 7 and processed for histochemical staining with Alcian blue and for gene expression analysis. As to be expected, palovarotene treatment inhibited the chondrogenic and chondrocyte phenotype as indicated by the significant drop in Alcian blue staining of the extracellular matrix compared with vehicle control cultures (Fig. 8A–E). This was accompanied by a steep drop in gene expression levels for *Sox9*, *Acan*, and *Col2a1* in treated versus control cultures (Fig. 8F). However, palovarotene treatment did not have obvious inhibitory effects on *Inhba* expression (Fig. 8G).

## Discussion

In line with our central hypothesis, the data in the present study demonstrate that palovarotene intersects with activin A along its path of therapeutic action against HO. We find that there is a substantial decrease in *Inhba*‐expressing/activin A‐producing cell populations present within the developing HO masses after palovarotene administration. In vehicle BMP2 controls, these *Inhba*‐expressing cell populations exhibit a committed chondrogenic and chondrocyte character, and, thus, their sharp decrease after palovarotene treatment is likely to be coupled causally to HO diminution. This interpretation is sustained by previous studies from this and other laboratories showing that activin A promotes chondrogenic cell differentiation.^(^ ^30^, ^62^ ^)^ A decrease in *Inhba*‐expressing cells could thus reduce chondrogenesis and, in turn, HO progression. It is of interest to note also that there was a clearly slower invasion of cells within the Matrigel scaffold and a paucity of cells reaching its deepest central region during palovarotene administration. It is not clear yet how palovarotene exerted such outcome, whether this response reflected a change in overall cell populations recruited during drug administration and/or whether the drug was able to directly affect cell migration or invasiveness. Although to be sorted out, these observations raise the more general issue as to how stem and progenitor cells normally sense and respond to tissue damage and inflammation and are recruited and/or guide themselves toward incipient HO sites to participate in its pathogenesis. A decrease in cell recruitment/migration could contribute to drug therapeutic potency. Considered together, our data indicate that palovarotene acts at several levels in its action against HO formation, inhibiting not only canonical BMP signaling^(^ ^17^, ^42^, ^63^ ^)^ and chondrogenesis^(^ ^16^, ^17^ ^)^ but also *Inhba*‐expressing cells participating in HO and, possibly, their recruitment. The data expand palovarotene's reach and action against HO pathogenic culprits and explain its effectiveness against it. We should note that there have been recent experimental advances in the identification of other potential HO targets. For example, Agarwal and colleagues found that the hypoxia factor Hif1α regulates mesenchymal cell recruitment and condensation during trauma‐induced HO and its pharmacologic inhibition dampens HO. Chen and colleagues observed that osteochondroprogenitors in mouse tendons require mTOR signaling for differentiation and that local pharmacologic inhibition of mTOR signaling suppressed trauma‐induced HO.^(^ ^64^ ^)^ A recent study identified the collagen cell surface receptor DDR2 (discoidin domain receptor 2) as a key regulator of mesenchymal cell behavior and HO in a mouse trauma model, and targeting it pharmacologically or genetically reduced HO.^(^ ^65^ ^)^ Altogether then, the HO research field continues to uncover important insights into HO pathogenesis and to point to effective possible therapeutic remedies.

The RARs are of central importance to development and physiology and regulate many processes at the cellular and molecular levels.^(^ ^21^ ^)^ Of note, ablation of retinyl aldehyde 2 (*Raldh2*)—the gene product turning natural inert cytoplasmic retinoid precursors into biologically active, RAR‐binding all‐*trans*‐retinoic acid—is incompatible with life and arrests embryonic development at the morula stage before gastrulation.^(^ ^66^ ^)^ As summarized above, the RARs are effective in regulating biological processes because they can act as transcriptional activators when associated with retinoic acid or other active ligands and as transcriptional repressors when unliganded.^(^ ^21^ ^)^ It is this fine‐tuned biology and its importance that have long attracted interest in using natural or synthetic retinoid agonists and antagonists to differentially modulate RAR function and exert desirable beneficial outcomes.^(^ ^67^ ^)^ An example of a natural clinically relevant retinoid is all‐*trans*‐retinoic acid, which is used to treat certain forms of leukemia, provoking tumor cell differentiation and inhibiting their cancerous character.^(^ ^68^ ^)^ Regarding synthetic retinoids, we recently showed that pathological premature growth plate closure in mice receiving a clinically relevant cancer drug can be prevented by systemic administration of a synthetic retinoid antagonist.^(^ ^69^ ^)^ An example of a recently FDA‐approved synthetic retinoid γ‐agonist is trifarotene, which is indicated for the treatment of acne.^(^ ^70^ ^)^ Synthetic retinoids are thus emerging as effective and possibly safer treatments for various disorders given their greater RAR specificity and selectivity compared with natural retinoids, eliciting fewer side effects and strengthening their clinical profile.^(^ ^67^ ^)^ Palovarotene was recently approved by HealthCanada for the treatment of FOP, and the data here explain in greater detail its therapeutic reach and action against HO.

Activin A was initially identified as a factor promoting the release of pituitary follicle stimulating hormone, hence its name.^(^ ^71^ ^)^ But over time, it has become apparent that activin A regulates many physiologic processes, including cell proliferation and differentiation, wound repair, and immune responses.^(^ ^54^ ^)^ Activin A is secreted as a dimer that is noncovalently bound to its pro‐domain, interacts with binding proteins and components that facilitate and/or limit its bioavailability including heparan sulfate‐rich proteoglycans and follistatin, and signals by interaction with cell surface type 1 and type 2 serine/threonine kinase receptors, including ALK4 and ALK7.^(^ ^72^ ^)^ We previously showed that there are three human activin A variants differing in their primary structure and exhibiting differential binding affinities for heparan sulfate, thus likely possessing diverse matrix interactive properties, distribution, and biological activities.^(^ ^73^ ^)^ Activin A roles in immunity have attracted much and well‐deserved attention.^(^ ^54^ ^)^ The protein is most notably produced by macrophages in which it exerts pro‐ or anti‐inflammatory effects depending on their activation status, and is also produced by cells including neutrophils and T cells.^(^ ^54^ ^)^ Activin A's roles in the musculoskeletal system were originally suggested by the finding that the protein is abundant in the bone matrix.^(^ ^74^ ^)^ It was then found that activin A promotes osteoblastogenesis,^(^ ^75^ ^)^ can influence the balance between that process and adipogenesis in marrow,^(^ ^76^ ^)^ and is required for chondrogenic differentiation of bone marrow mesenchymal progenitor cells.^(^ ^62^ ^)^ More recent studies demonstrating activin A's roles in promoting genetic and acquired HO are thus very much in line with those previous findings.^(^ ^27^, ^30^ ^)^ Given the complex nature of activin A biology, it is possible that the protein may exert equally complex roles in HO pathogenesis, including participating in the initial inflammatory phase as a product of macrophages and then promoting chondrogenic differentiation and chondrocyte function. Thus, targeting activin A to restrain or even prevent HO is based on solid ground. The data here provide strong evidence that by directly or indirectly dampening activin A‐producing cells present at HO forming sites, palovarotene can mitigate activin A action and in turn counteract yet one more culprit in HO pathogenesis.

It is important to note the limitations of the present study. The subdermal Matrigel/BMP implantation model used here is a popular and well‐established approach to study HO but does and cannot reproduce clinical HO in its entirety. This is an experimental conundrum that applies to the several other experimental HO models created in several animal species over the years.^(^ ^2^ ^)^ Thus, data generated here need to be considered and evaluated with caution. Our study has focused on the initial phases of the HO process, with the aim of capturing the processes and mechanisms that set the overall pathogenesis in motion. Later time points, and particularly those during which bone and marrow develop, would need to be examined in the future. We have not observed a direct effect of palovarotene on *Inhba* gene expression as we had expected to find. It is possible that the cell populations used in these in vitro studies may not contain direct targets of drug action, and if so, the study would need to be extended to other and diverse populations in the future to characterize gene sensibility and responses.

## Author Contributions

**Christina Mundy:** Conceptualization; data curation; formal analysis; investigation; writing – review and editing. **lutian yao:** Conceptualization; data curation; formal analysis; investigation; writing – review and editing. **Kelly A. Shaughnessy:** Data curation; formal analysis; methodology; validation. **Cheri Saunders:** Formal analysis; investigation; methodology; validation. **Eileen M. Shore:** Conceptualization; formal analysis; writing – review and editing. **Eiki Koyama:** Data curation; formal analysis; investigation; validation; visualization. **Maurizio Pacifici:** Conceptualization; data curation; formal analysis; funding acquisition; supervision; writing – original draft.

### Peer Review

The peer review history for this article is available at https://www.webofscience.com/api/gateway/wos/peer‐review/10.1002/jbm4.10821.

## Disclosures

All authors state that they have no conflicts of interest.

## Supporting information


**Fig. S1.** Violin plot of expression of immune cell marker genes.

**Fig. S2.** Gene set variation analysis (GSVA) of KEGG pathway database and Hallmark gene sets for cell clusters in day 5 HO samples. Note that the Vehicle group displayed a significant and distinct upregulation of glycolysis and hypoxia pathways known to be important for chondrogenesis that are markedly reduced after Palovarotene administration (yellow box area). Note also that inflammatory pathways were particularly prominent in immune cell clusters (upper right).

**Fig. S3.** CellChat signaling analysis of putative interactions amongst cell populations present in the HO masses delineated in Fig. 3. Only 8 cell groups are shown here because clusters 0, 1, 4 and 10 from Fig. 3 – all representing macrophages largely – were combined into a single population. (*A, B*) Palo group is predicted to exhibit a significant decrease in interactions amongst populations including mesenchymal 1 and mesenchymal 2/skeletogenic cells, possibly reflecting drug's ability to dampen cartilage formation. (*C*) Left panel: Comparison of signaling from Mesenchymal/Skeletogenic to other cell populations. Right panel: Comparison of signaling from other cell populations to Mesenchymal/Skeletogenic cluster. Blue color indicates a decreased number in interactions in Palo treatment group compared with Vehicle. Red color indicates an increased number of interactions in Palo treatment group compared with Vehicle.

**Table S1.** Sequences of the primers used to determine expression of chondrogenic markers and genes.

**Table S2.** Number and percentage of different cell types in 3 conditions.

**Table S3.** Number and percentage of different cell types of mesenchymal lineage in 3 conditions.
