# Supplementary figures and images for: Palovarotene Action Against Heterotopic Ossification Includes a Reduction of Local Participating Activin A‐Expressing Cell Populations
Source: JBMR Plus. 2023 Oct 19;7(12):e10821. doi: 10.1002/jbm4.10821 (PMC10731142; doi:10.1002/jbm4.10821)

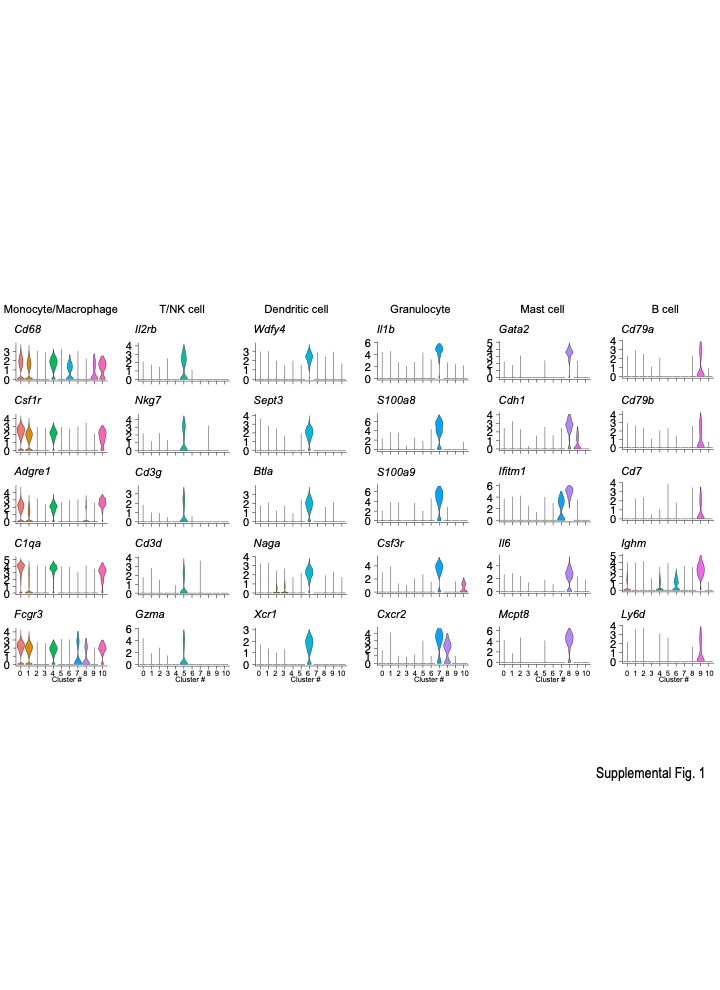

Supplement: Supplementary file 1 — Fig. S1. Violin plot of expression of immune cell marker genes. [file JBM4-7-e10821-s003.tiff]

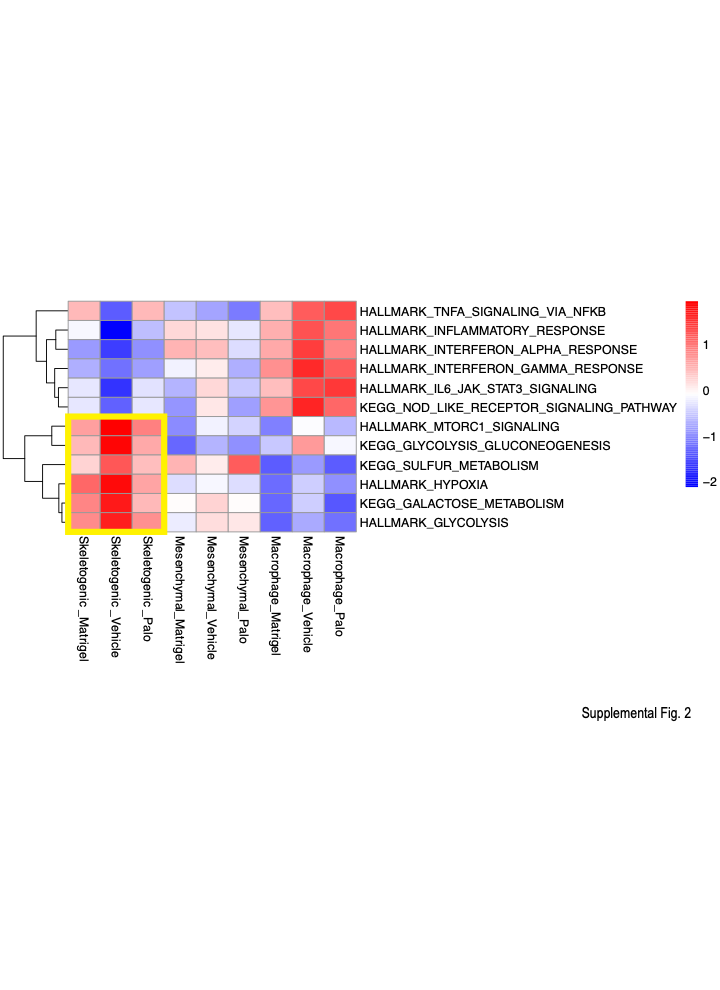

Supplement: Supplementary file 2 — Fig. S2. Gene set variation analysis (GSVA) of KEGG pathway database and Hallmark gene sets for cell clusters in day 5 HO samples. Note that the Vehicle group displayed a significant and distinct upregulation of glycolysis and hypoxia pathways known to be important for chondrogenesis that are markedly reduced after Palovarotene administration (yellow box area). Note also that inflammatory pathways were particularly prominent in immune cell clusters (upper right). [file JBM4-7-e10821-s006.tiff]

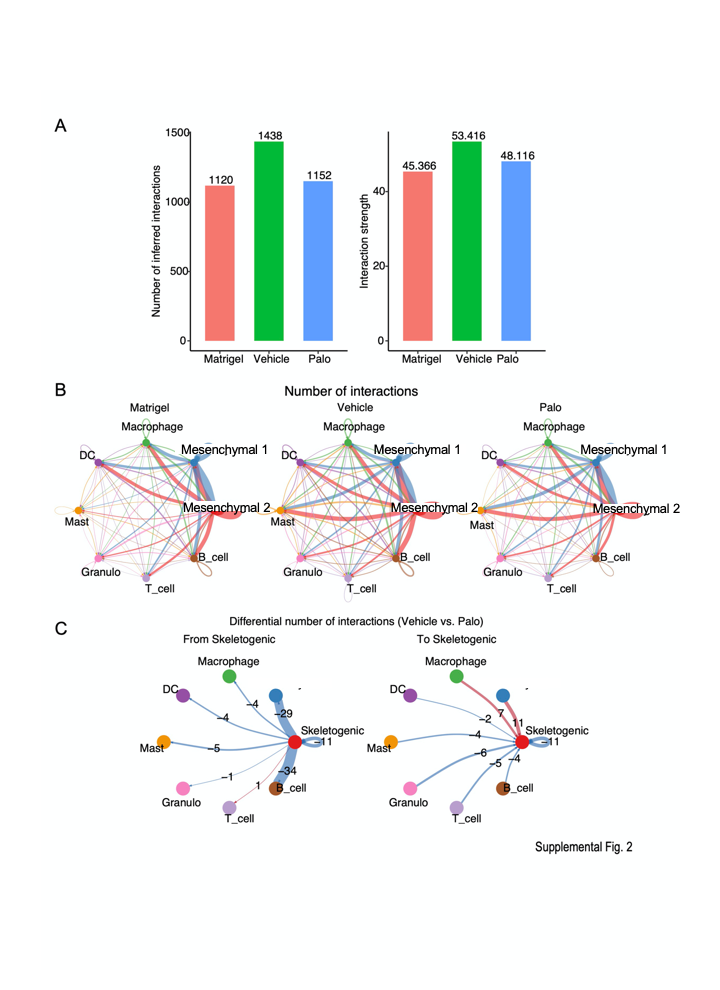

Supplement: Supplementary file 3 — Fig. S3. CellChat signaling analysis of putative interactions amongst cell populations present in the HO masses delineated in Fig. 3. Only 8 cell groups are shown here because clusters 0, 1, 4 and 10 from Fig. 3 – all representing macrophages largely – were combined into a single population. (A, B) Palo group is predicted to exhibit a significant decrease in interactions amongst populations including mesenchymal 1 and mesenchymal 2/skeletogenic cells, possibly reflecting drug's ability to dampen cartilage formation. (C) Left panel: Comparison of signaling from Mesenchymal/Skeletogenic to other cell populations. Right panel: Comparison of signaling from other cell populations to Mesenchymal/Skeletogenic cluster. Blue color indicates a decreased number in interactions in Palo treatment group compared with Vehicle. Red color indicates an increased number of interactions in Palo treatment group compared with Vehicle. [file JBM4-7-e10821-s002.tiff]
