# Supplementary material for: Palovarotene Action Against Heterotopic Ossification Includes a Reduction of Local Participating Activin A‐Expressing Cell Populations
Source: JBMR Plus. 2023 Oct 19;7(12):e10821. doi: 10.1002/jbm4.10821 (PMC10731142; doi:10.1002/jbm4.10821)
Supplement: Supplementary file 4 — Table S1. Sequences of the primers used to determine expression of chondrogenic markers and genes. [file JBM4-7-e10821-s004.docx]

**Table S1.** Sequences of the primers used to determine expression of chondrogenic markers and genes.

| Gene | Accession No. | Forward Sequence (5’-3’) | Reverse Sequence (5’-3’) | Size (bp) |
| --- | --- | --- | --- | --- |
| *Acan* | NM_007424 | ggagcgagtccaactcttca | cgctcagtgagttgtcatgg | 120 |
| *Col2a1* | NM_031163 | ctacggtgtcagggccag | gtgtcacacacacagatgcg | 116 |
| *Sox9* | NM_0011448 | gagctcagcaagactctggg | cggggctggtacttgtaatc | 131 |
| *Rn18s* | NR_003278 | gcaattattccccatgaacg | ggcctcactaaaccatccaa | 113 |
