# Supplementary material for: Palovarotene Action Against Heterotopic Ossification Includes a Reduction of Local Participating Activin A‐Expressing Cell Populations
Source: JBMR Plus. 2023 Oct 19;7(12):e10821. doi: 10.1002/jbm4.10821 (PMC10731142; doi:10.1002/jbm4.10821)
Supplement: Supplementary file 5 — Table S2. Number and percentage of different cell types in 3 conditions. [file JBM4-7-e10821-s005.docx]

|  | Matrigel | | Vehicle | | Palo | |
| --- | --- | --- | --- | --- | --- | --- |
|  | Cell # | Cell % | Cell # | Cell % | Cell # | Cell % |
| 0 Macrophage | 3974 | 45.5% | 2153 | 39.3% | 3463 | 47.5% |
| 1 Macrophage | 2039 | 23.4% | 1289 | 23.5% | 2031 | 27.8% |
| 2 Mesenchymal | 1146 | 13.1% | 1286 | 23.5% | 866 | 11.9% |
| 3 Mesenchymal | 952 | 10.9% | 132 | 2.4% | 334 | 4.6% |
| 4 Macrophage | 303 | 3.5% | 67 | 1.2% | 295 | 4.0% |
| 5 T/NK cell | 146 | 1.7% | 297 | 5.4% | 97 | 1.3% |
| 6 Dendritic cell | 141 | 1.6% | 40 | 0.7% | 94 | 1.3% |
| 7 Granulocyte | 3 | 0.0% | 85 | 1.6% | 15 | 0.2% |
| 8 Mast cell | 1 | 0.0% | 95 | 1.7% | 0 | 0.0% |
| 9 B cell | 21 | 0.2% | 30 | 0.5% | 28 | 0.4% |
| 10 Macrophage | 2 | 0.0% | 2 | 0.0% | 73 | 1.0% |
|  |  |  |  |  |  |  |

**Table S2.** Number and percentage of different cell types in 3 conditions.
