# Supplementary material for: Palovarotene Action Against Heterotopic Ossification Includes a Reduction of Local Participating Activin A‐Expressing Cell Populations
Source: JBMR Plus. 2023 Oct 19;7(12):e10821. doi: 10.1002/jbm4.10821 (PMC10731142; doi:10.1002/jbm4.10821)
Supplement: Supplementary file 6 — Table S3. Number and percentage of different cell types of mesenchymal lineage in 3 conditions. [file JBM4-7-e10821-s001.docx]

**Table S3.** Number and percentage of different cell types of mesenchymal lineage in 3 conditions.

|  | Matrigel | | Vehicle | | Palo | |
| --- | --- | --- | --- | --- | --- | --- |
|  | Cell # | Cell % | Cell # | Cell % | Cell # | Cell % |
| 0 MPC | 781 | 44.7% | 81 | 6.3% | 284 | 26.7% |
| 1 Intermediate | 184 | 10.5% | 66 | 5.2% | 60 | 5.6% |
| 2 Chondrogenic | 466 | 26.7% | 725 | 56.6% | 460 | 43.2% |
| 3 Fibroblast-like | 313 | 17.9% | 175 | 13.7% | 261 | 24.5% |
| 4 Chondrocyte | 4 | 0.2% | 234 | 18.3% | 0 | 0.0% |
|  |  |  |  |  |  |  |
